# Radioligand therapy (RLT) used to treat cardiac metastasis of pancreatic neuroendocrine tumor

**DOI:** 10.1002/ccr3.8622

**Published:** 2024-03-05

**Authors:** Kieran J. Ved, Arjun Bhatt, Aidan M. Burke, Michael C. Larkins, Dishita Pandya, Constantin B. Marcu

**Affiliations:** ^1^ Brody School of Medicine, East Carolina University Greenville North Carolina USA; ^2^ Department of Radiation Oncology, Brody School of Medicine East Carolina University Greenville North Carolina USA; ^3^ Department of Cardiovascular Sciences, Brody School of Medicine East Carolina University Greenville North Carolina USA

**Keywords:** cardiac metastases, Lutathera, neuroendocrine neoplasms (NENs), peptide receptor radiotherapy (PRRT), somatostatin receptor

## Abstract

Radioligand Therapy (RLT) in the form of [177Lu] Lu‐DOTA‐TATE (Lutathera®) is a promising treatment for pancreatic neuroendocrine tumors (pNETs) with cardiac metastasis. We present a patient treated with [177Lu] Lu‐DOTA‐TATE that showed shrinkage of metastasis after four treatments at 7.4 GBq every 8 weeks.

## 1 INTRODUCTION

Neuroendocrine neoplasms (NEN, formerly known as Neuroendocrine Tumors, or NET) are a diverse group of neoplasms arising from so‐called neuroendocrine cells and can occur almost everywhere in the body.^1^, ^2^ NEN are mostly found in the gastrointestinal (GI) tract and lung, however they have been reported in the pancreas as well; prevalence and incidence are variable dependent on organ site.^1^ Pancreatic neuroendocrine tumors (pNET) have an incidence of about 0.43 per 100,000 people based on data in the National Cancer Institute Surveillance, Epidemiology, and End Results (SEER) registry, and comprise about 1%–2% of all pancreatic neoplasms.^3^ The incidence has reportedly more than doubled in the last 20–30 years secondary to increased physician awareness and improvements in the quality and frequency of clinical imaging. Autopsy studies have found a range of pNET prevalence, from 0.8% to 10%, with the implication that most pNET are clinically silent. Recent analysis suggests that pNET arise from pluripotent stem cells in the pancreatic ductal/acinar system.^4^ 90% of pNET arise sporadically, with the remaining 10% arising in patients with an underlying genetic syndrome such as multiple endocrine neoplasia type I (MEN1).^3^

The diagnosis of pNET begins with a detailed history and physical exam to assess for signs of mass effects, metastasis, endocrine symptoms, and family history.^3^ Numerous biomarkers are available to aid in the diagnosis of pNET, including but not limited to Chromogranin A, insulin, glucagon, and vasoactive intestinal peptide (VIP).^5^ These biomarkers in addition to novel biomarkers such as circulating tumor cells, have various sensitivities and specificities for the diagnosis of pNET. Imaging plays a crucial role in the localization and staging of pNET, with computed tomography (CT) being the most common. Other imaging modalities such as magnetic resonance, radiolabeled somatostatin analogs, and positron emission tomography (PET) are used as well.^3^ Staging of pNET falls under two common staging systems: the American Joint Committee on Cancer (AJCC) 8th edition^6^ and the European Neuroendocrine Tumor Society (ENETS), which proposes separate guidelines for functioning^7^ and nonfunctioning^8^ tumors.

The mainstay treatment of pNET is surgical resection, as this generally is the only potentially curative treatment.^3^ Numerous surgical methods exist, ranging from simple enucleation for small lesions (with more recent literature suggesting the use of this method with mainly insulinomas) to complete oncologic resection with or without splenectomy and pancreaticoduodenectomy. Watchful waiting is also an option in patients with lesions <2 cm.^6^, ^7^, ^8^ Laparoscopic resection (so‐called minimally invasive surgical treatment) is being increasingly implemented to limit blood loss and operative time. Advanced disease may still benefit from surgical treatment in the form of cryoreductive surgery and the treatment of liver metastases.

Medical modalities are available for the management of the symptoms of pNET and more recently to co‐treat pNET along with surgery.^3^ Managing excess hormonal secretions (e.g., with proton pump inhibitors in patients with Zollinger‐Ellison syndrome, etc.) and using somatostatin analogs such as lantreotide to target overexpressed somatostatin receptors (SSTR) are two examples. Chemotherapy, such as with cisplatin and etoposide, has shown benefit, though the chemotherapeutic agent recommended varies based on disease Grade.

The most recent development in the medical treatment of pNET is radioligand therapy (RLT), in which is performed by attaching radioactive isotopes to somatostatin analogs to selectively deliver radiotherapy to tumor cells.^3^ One such agent, [177Lu] Lu‐DOTA‐TATE (Lutathera®), was approved by the US Food and Drug Administration in 2018 for the treatment of adults with SSTR‐positive gastropancreatic NEN and is the most widely‐used RLT.^9^ RLT is generally well‐tolerated, with rare complications that include carcinoid crisis, bone marrow suppression, and the development of myelodysplastic syndrome or acute myeloid leukemia. While initially approved as a one‐time treatment in patients with unresectable or metastatic disease, recent literature has focused on the re‐treatment of patients with RLT.

It is rare to find myocardial metastasis from NEN, with an overall incidence of 4% among metastatic carcinoid patients.^10^ Cardiac metastases are typically asymptomatic at presentation and detected incidentally. The rarity of cardiac NEN metastases makes it challenging to establish standardized treatment protocols. Review of the literature revealed three case reports^10^, ^11^, ^12^ and one case series^13^ of patients with cardiac metastases secondary to neuroendocrine tumors; only one patient of these five was treated with [177Lu] Lu‐DOTA‐TATE for cardiac metastasis, and achieved a stable disease state for 20 months after administration before ultimately passing away.^13^ This case describes a patient diagnosed with pNET metastatic to the left ventricular myocardium who was treated with [177Lu] Lu‐DOTA‐TATE, ultimately showing both reduction in tumor size on cardiac MRI and reduction in uptake via PET.

## 2 CASE HISTORY

We describe a 75‐year‐old female with past medical history notable for melanoma. Family history was notable for multiple immediate family members with GI cancers (father with colon cancer, brother with esophageal cancer). She was noted to be in clinical remission for more than 18 years after diagnosis, neoadjuvant chemotherapy, and surgical resection of her melanoma. She followed with a dermatologist regularly and was following skin protective measure daily. Eleven years after her melanoma diagnosis and treatment, she presented with abdominal distension and mid‐epigastric abdominal pain and was found to have a 9 cm mass in the head of her pancreas, subsequently found to be a neuroendocrine tumor on biopsy. Patient's disease was noted as well‐differentiated, unifocal, with <2 cells with mitotic activity per 10 under high‐powered field. Two of four lymph nodes were positive, and the patient was staged as pT3, pN1. Shortly thereafter she underwent pancreaticoduodenectomy with negative margins. Within 2 years she developed metastatic recurrence in the left lobe of her liver and underwent lanreotide treatment for the next 3 years. In the interim she developed breast cancer (Stage IA of the right breast: cT1c, cN0, cM0, Grade I; ER/PR positive, HER2 negative) and underwent lumpectomy, adjuvant radiotherapy, and maintenance endocrine therapy (anastrozole).

## 3 METHODS

She went on to develop widespread metastatic disease after 3 years of once monthly lanreotide treatment with involvement of her liver, heart, lungs, and abdominal viscera, as evidenced via PET (using gallium (Ga)‐68 DOTATATE as the contrast medium). Two to four areas focal areas in the patient's liver and two small nodular densities in the upper abdomen (Krenning scores of four for both) were identified, as was a focus of discrete activity partially superimposed on the patient's left ventricle (Krenning score of three with a standardized uptake value, or SUV, of 27.4). The patient was consulted to the cardiology service and discussed at tumor board. Lanreotide dosing was increased to once every 2 weeks while clinical decision‐making was under way based on data from the CLARINET FORTE clinical trial.^14^

**FIGURE 1:**
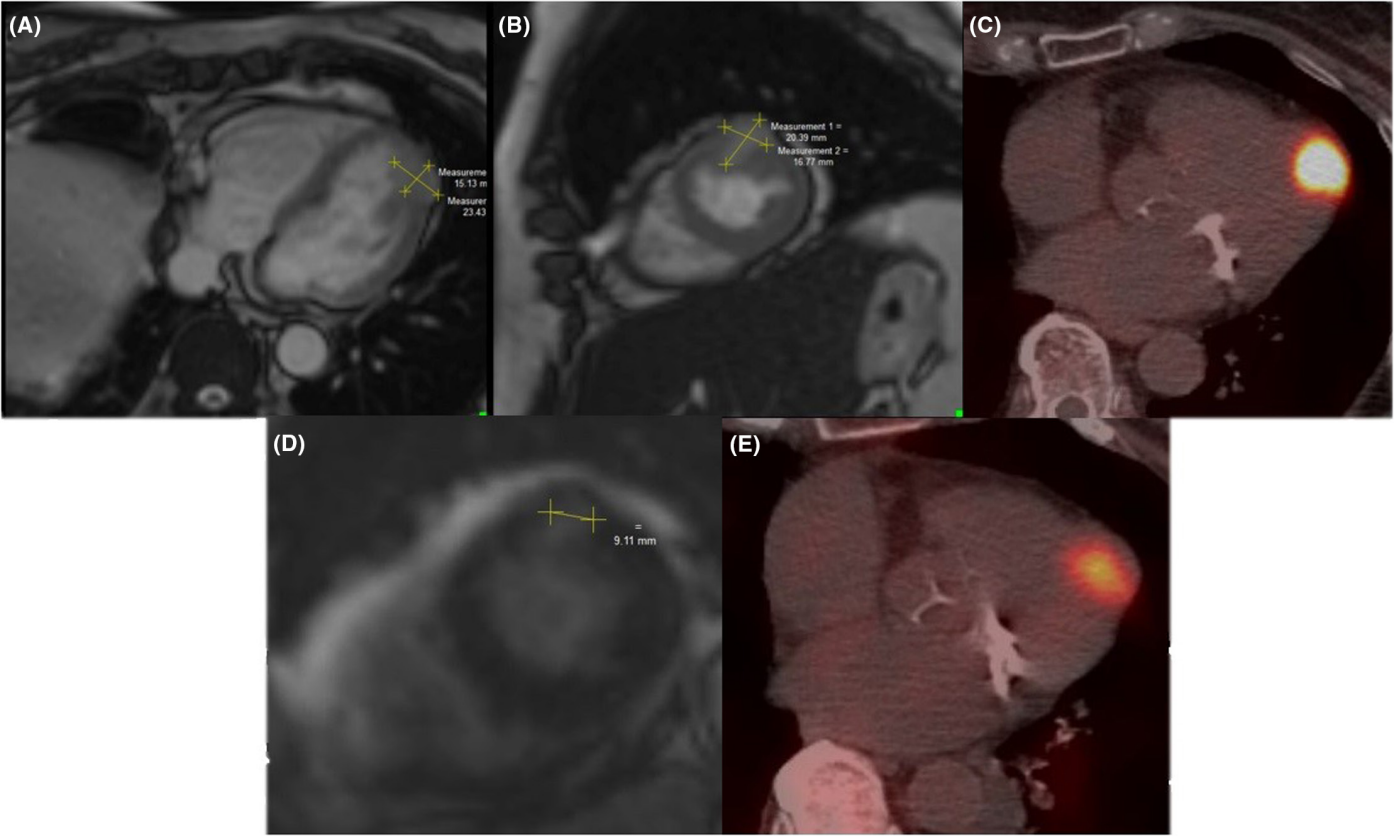
Imaging of a patient with metastatic pNET to the heart, treated with [177Lu] Lu‐DOTA‐TATE and lanreotide. (A, B) Cardiac magnetic resonance imaging (cMRI) with gadolinium (Gd) contrast showing our patient's cardiac metastases prior to treatment. The mass measured 15 mm x 23 mm and did not impact cardiac function (ejection fraction was estimated as 75%). Short axis (A) and axial (B) orientations are shown. (C) Positron emission tomography (PET) imaging with gallium (Ga)‐68 contrast taken prior to patient treatment showing hyperintensity at the same location as the mass seen in Figure 1A, B; intensity was measured at 27.4 standardized uptake units (SUV). (D) cMRI showing decreased lesion size compared to Figure 1A, B (now about 9 mm in diameter at widest point). (E) PET imaging after treatment with [177Lu] Lu‐DOTA‐TATE showing decreased uptake (now 24.8 SUV) compared to Figure 1C.

Concerning the patient's potential cardiac metastasis, assessment of the functional impact was attempted by transthoracic echocardiography which showed normal left ventricular systolic function with an ejection fraction (EF) of 65% with no thickening of the heart or abnormal heart strain but did not visualize the suspected cardiac metastases well. Follow‐up cardiac magnetic resonance imaging (MRI) with gadolinium (Gd)‐contrast demonstrated an oval‐shaped, Gd contrast‐enhancing mass (15 mm × 23 mm) at the border between the mid and distal anterior/lateral segments of the left ventricle, consistent with a pNET metastasis with a necrotic core (see Figure 1A,B; Figure 1C shows the uptake on PET imaging). The left ventricle EF was estimated at 75%; the left ventricle was noted as being of normal size with hyperdynamic motion. A cardiac biopsy was deferred, as it was felt to be technically challenging given the lesion size and location. After discussion of the patient at a multi‐disciplinary tumor board and shared decision making, it was decided that approval for RLT in the form of [177Lu] Lu‐DOTA‐TATE (Lutathera®) would be sought.

After 3 months of high‐dose lanreotide the patient was approved for [177Lu] Lu‐DOTA‐TATE, and treatment was started at 7.4 GBq every 8 weeks, as well as maintenance lanreotide every 4 weeks. She completed her initial course of four infusions of [177Lu] Lu‐DOTA‐TATE uneventfully. Several weeks after completion of treatment, the patient was hospitalized for 14 days due to COVID‐19 infection and altered mental status; imaging showed evidenced of multiple infarcts in the patient's brain, liver, and spleen. Agitated saline transthoracic echocardiogram study which demonstrated no evidence for intracardiac shunting and no obvious cardiac source of her emboli. It was recommended she wear an event monitor as an outpatient to rule out atrial fibrillation. Her workup overall for etiology of her hypercoagulable/embolic state was attributed to her COVID‐19 infection and malignancy, and she was anticoagulated with enoxaparin. One set of blood cultures grew *Streptococcus anginosus* which was presumed to be a contaminant; however, given her emboli and immunocompromised status, she was started on and completed a two‐week course of ceftriaxone. She was discharged to rehab and continued on her maintenance lanreotide. Her event monitor did not capture any evidence of atrial fibrillation.

## 4 CONCLUSION AND RESULTS

Following discharge, repeat cardiac MRI with Gd contrast showed no evidence of left ventricular thrombus. There was, however, a significant decrease in the size of the distal anterolateral cardiac metastasis without other obvious cardiac progression of disease (see Figure 1D). Repeat Ga‐68 DOTATATE PET demonstrated interval slight decreased level of activity of cardiac metastases relative to prior scans (24.8 SUV, compared to 27.4 SUV on previous PET; see Figure 1E compared to Figure 1C). The patient remained on monthly lanreotide injections and monitored for response to RLT. Two months after hospital discharge, she was admitted to an outside institution and diagnosed and treated for septic shock. She ultimately expired due to circulatory collapse secondary to shock.

## 5 DISCUSSION

Cardiac metastases from NEN are a relatively rare phenomenon per the literature, with an overall incidence of 4%.^10^ Review of the literature showed only three case reports^10^, ^11^, ^12^ and one case series^13^ regarding treatment of patients with cardiac metastases secondary to neuroendocrine tumors. The case series, Makis et al., came about as a review of 251 patients at a single institution with neuroendocrine tumors; two of these patients had cardiac metastasis (incidence of 0.8%) and one of these two patients was ultimately treated with [177Lu] Lu‐DOTA‐TATE. This patient achieved disease stability for 20 months before expiring due to failure to thrive. In contrast, the patient we describe in our case report received four doses of [177Lu] Lu‐DOTA‐TATE over 8 months; shortly after completion of their RLT they contracted COVID‐19 and developed diffuse coagulation, thought to be secondary to the COVID infection but also in the context of malignancy. No cardiac source was found for the emboli and no thrombus was ever noted in the patient's cardiac chambers. They ultimately expired within 3 months of completion of RLT after being admitted and treated at an outside institution for septic shock, though not before showing some improvement in disease, both with shrinkage of cardiac metastasis and with decreased uptake on PET imaging.

Our patient's initially asymptomatic presentation following cardiac metastasis was in keeping with the literature.^10^ The patient's initial disease progression (from 9 cm pNET in the head of the pancreas to metastases) was thought to be in the context of stopped lanreotide injections, which were halted for multiple months as the patient showed no disease progression following initial surgery and chemotherapy. While our patient's cardiac metastasis was never confirmed to stem from pNET via biopsy (this was deferred by the interventional radiology service as it was considered too technically challenging), given our patient's near two‐decade history of clinically‐stable melanoma and close follow‐up with a dermatologist without evidence of disease progression, all involved specialties considered the patient's cardiac metastasis to stem from her initial pNET.

Our patient did undergo genetic testing given her personal history of multiple cancers and family history of GI cancers: her brother was diagnosed with esophageal cancer, her father with colon cancer, her paternal grandmother with kidney cancer, and her paternal great grandmother with abdominal cancer. The details of these specific cancers are not known. Genetic testing revealed a variant of unknown significance in the tuberous sclerosis complex (TSC)2 gene, potentially pointing to an etiology for her pNET. However, she had no history of hamartomas, seizures, or intellectual disability, as would be expected in patients with tuberous sclerosis.^15^

The decision to implement [177Lu] Lu‐DOTA‐TATE was made given the severity of the patient's disease, though high‐dose (one dose every 2 weeks) lanreotide was used in the interim between the decision to implement [177Lu] Lu‐DOTA‐TATE and insurance approval. Due to their rarity, no guidelines regarding treatment of cardiac metastases from NEN exist, though guidelines for the primary NEN are spelled out in both the AJCC (8th edition)^6^; and ENETS guidelines for functioning and nonfunctioning NEN.^7^, ^8^ Our case adds to the growing body of evidence of the efficacy of [177Lu] Lu‐DOTA‐TATE for treatment of patients with metastatic disease.

We describe a patient with rare cardiac metastases secondary to pNET treated with [177Lu] Lu‐DOTA‐TATE in conjunction with lanreotide. Our patient showed evidence of tumor shrinkage and decreased uptake on cardiac MRI and PET imaging, respectively. This case is one of only a handful to report on cardiac metastases in the context of NEN and with treatment via [177Lu] Lu‐DOTA‐TATE.

## AUTHOR CONTRIBUTIONS

**Kieran J. Ved:** Conceptualization; project administration; writing – original draft; writing – review and editing. **Arjun Bhatt:** Conceptualization; project administration; visualization; writing – review and editing. **Aidan M. Burke:** Conceptualization; investigation; supervision; writing – review and editing. **Michael C. Larkins:** Supervision; writing – review and editing. **Dishita Pandya:** Supervision; visualization; writing – review and editing. **Constantin B. Marcu:** Data curation; project administration; resources; supervision; writing – review and editing.

## CONSENT

Written informed consent was obtained from the patient to publish this report in accordance with the journal's patient consent policy.

## Data Availability

Data sharing not applicable to this article as no datasets were generated or analyzed during the current study.
